# First detection of *Leishmania major* in dogs living in an endemic area of zoonotic cutaneous leishmaniasis in Tunisia

**DOI:** 10.1186/s13071-024-06395-2

**Published:** 2024-08-09

**Authors:** Maria Paola Maurelli, Lilia Zribi, Nour El Houda Ben Fayala, Valentina Foglia Manzillo, Ines Balestrino, Noureddine Hamdi, Aida Bouratbine, Manuela Gizzarelli, Laura Rinaldi, Karim Aoun, Gaetano Oliva

**Affiliations:** 1 Department of Veterinary Medicine and Animal Production, University of Naples “Federico II”, Naples, Italy; 2 Institut Pasteur de Tunis, Tunis, Tunisia; 3 Regional Commissariat for Agricultural Development-Ministry of Agriculture (CRDA), Kairouan, Tunisia

**Keywords:** *Leishmania major*, Dogs, Leishmaniosis

## Abstract

**Background:**

Dogs are considered the main domestic animals that may be a reservoir for *Leishmania infantum*, the agent of zoonotic visceral leishmaniasis (ZVL) in several countries of the world. The dog may host other *Leishmania* species, but its epidemiological role in the maintenance and spreading of these parasites is not completely elucidated. Zoonotic cutaneous leishmaniasis (ZCL), caused by *Leishmania major*, affects thousands of people every year and is particularly diffused in many countries of North Africa and Middle East Asia. In ZCL endemic countries, few reports of *L. major*-positive dogs have been reported, probably because most human cases occur in poor rural areas where the social role of the dog and its medical management is not well considered. The aim of the present study is to better understand the possible involvement of domestic dogs in the epidemiology of ZCL.

**Methods:**

Our research focused on a well-established endemic focus of ZCL, in the area of Echrarda, Kairouan Governorate, central Tunisia. A total of 51 dogs with no or mild clinical signs of vector borne diseases were selected in small villages where human cases of ZCL are yearly present. All dogs were sampled for the *Leishmania* spp. diagnosis, by using the following procedures: blood sample for serology and buffy coat quantitative polymerase chain reaction (qPCR), popliteal fine needle aspiration, and cutaneous biopsy punch for lymph node and skin qPCR.

**Results:**

The results demonstrated a high percentage (21.6%) of dogs positive at least at one or more test; the most sensitive technique was the lymph node qPCR that detected 8/11 positive dogs. Nine, out of the eleven positive dogs, resulted as infected by *Leishmania infantum*; ITS1-PCR-sequencing allowed *Leishmania major* identification in the remaining two cases, both from the popliteal lymph node samples, which can suggest a possible visceral spread of a cutaneous *Leishmania* species in the dog. Interestingly, one of the two *L. major*-positive dogs was living in the same house where 6-year-old children showed cutaneous lesions referred to as ZCL.

**Conclusions:**

To our knowledge, this is the first report of *L. major*-positive dogs in Tunisia, the  epidemiological role of which remains under investigation.

**Graphical Abstract:**

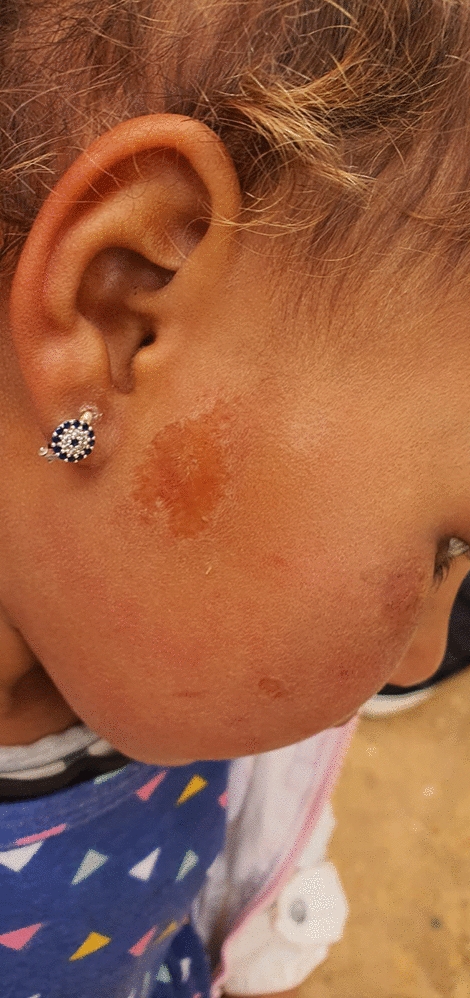

## Background

Tunisia represents a perfect example of a Mediterranean Country in which different *Leishmania* species may express their infectivity. Visceral leishmaniasis (VL) caused by *Leishmania* (*L*.) *infantum* and zoonotic cutaneous leishmaniasis (ZCL) caused by *L*. *major* are endemic, while chronic cutaneous leishmaniasis (CCL) caused by *L. tropica* is considered confined in specific foci of interest involving the south–east and south-west of the country. Interestingly, the two most important cutaneous *Leishmania* species, *L. major* and *L. tropica* may overlap in some areas of the Tunisia [1, 2]. Likewise, visceral and ZCL endemic foci overlap was identified in some northern central districts also if they remain geographically distinct [3, 4]. The role of different animals in the epidemiological transmission of these *Leishmania* species is continually under revision. *L. major* is transmitted by the sand fly vector *Phlebotomus papatasi* [5], with *Psammomys obesus* and *Meriones shawi* considered as the principal reservoir hosts [6]. *Mustela nivalis*, *Paraechinus aethiopicus*, *Atelerix algirus*, *Ctenodactylus gundi*, and *Psammomys vexillaris* are considered as potential reservoirs for *L. major* [7]. Dogs undebatably play a pivotal role in many situations, where the transmission cycle of *L. infantum* occurs while its contribution to the life cycle of *L*. *major* is considered not relevant despite the fact that this species has been detected in very few cases in dogs [8, 9]. *L. major* was first identified from an ear ulcer of a dog in Saudi Arabia [10] and from the spleen and blood of two dogs from Egypt [11]. The first clinical report was described in Israel [8], but because of the scarcity of clinical demonstrations, there are no definitive indications for the clinical features nor for the treatment. The infection has also been reported during some epidemiological studies performed in Middle East countries, such as Saudi Arabia, Iran, Iraq, Turkey, and recently in Burkina Faso [12, 13], while a previous study performed in a neighboring area of Tunisia did not allow for the identification of this species in dog [6]. The role of the dog in sustaining the transmission of *L. major* to humans remain under investigation. The aim of the present study is to assess the presence of *L. major* infected dogs in Tunisia to add information on the potential involvement of this domestic animal in the ZCL epidemiology, by focusing the research in a restricted area considered a stable endemic focus of ZCL.

### Methods

The study was performed in small built-up areas belonging to the district of Echrarda (35 ° 07′ 08’’ North, 10 ° 01′ 49’’ East), the southern part of Kairouan Governorate, Tunisia. The selection of a small cluster of houses that hosted dogs was based on the knowledge of recent ZCL confirmed diagnoses, while in the whole district an incidence of 96.7 cases/100,000 Ha was reported [14]. The area is characterized by a rural, semi-arid environment. The registered population is 27,518 (2014) on 330 km^2^, while there is scarce information on the dogs’ population. A total of 51 dogs, 31 males and 20 females, with an estimated age ranging between 1 and 12 years were recruited on the basis of the compliance of the owners to join the anti-rabies vaccination campaign. After the vaccine administration, the owners were informed about the possibility to submit the dogs to further sampling for *Leishmania* spp. diagnosis, informed consent was obtained from all participants. Dogs were submitted to clinical examination by filling a clinical form and sampled for blood, popliteal lymph node (LN) aspirate, skin punch biopsy and conjunctival swab. Blood samples (3 mL) were obtained by peripheral veins, and then divided into two aliquots, respectively, in empty and EDTA-coated tubes for serum, plasma and buffy coat (BC) collection. Lymph node aspiration was performed by one of the two popliteal lymph nodes, and skin biopsy was obtained by punching the surface between neck and ear,with a 1 mm punch biopsy. Conjunctival swabs were performed by both lower eyelids but processed together. The collected materials were stored in 1.5 mL tubes. Sample collection was performed following the Good Clinical Practice medical procedures, in accordance with the international guidelines for animal welfare. Tubes were kept at 4 °C until the arrival at laboratory. Blood was centrifuged 10 min at 2500 rpm to separate red cells, BC and plasma. Plasma was stored at −20 °C until serological analysis; BC, LN aspirates, skin biopsy samples and conjunctival swabs were stored at −20 °C until DNA extraction. Serological diagnosis was performed by enzyme-linked immunosorbent assay (ELISA) ID Screen Leishmaniasis Indirect Test^®^ kit (ID vet, Innovative diagnostics, France), the same used in a previous study [6]. Briefly, optical densities were read at 450 nm (ELISA plate reader Anthos^®^, Bristol, England). Results that were expressed as percentages of plasma with results between 40% and 50% and designated by the manufacturer as “doubtful” were tested by an indirect immunofluorescence antibodies test (IFAT) using spot slides sensitized by *L. infantum* promastigotes; a threshold of 1:100 defined seropositivity. Additional serological testing was performed on *Leishmania* spp. samples to detect possible co-infections with other canine vector borne diseases (CVBDs). Antibodies to *Anaplasma phagocytophilum*/*Anaplasma platys*, *Borrelia burgdorferi*, *Ehrlichia canis*/*Erlichia ewingii*, and antigens to *Dirofilaria immitis* were detected by the SNAP 4Dx Plus test (IDEXX Laboratories, Westbrook, Maine, US).

DNA was extracted from 51 buffy coat, lymph node and skin biopsy samples using the DNeasy Blood and Tissue kit (Qiagen, Leipzig, Germany) according to the manufacturer’s instructions. Moreover, DNA was extracted from 51 conjunctival swabs, using the Leishmania Screen Glow (Avantech Group, Angri, Italy) following the protocol described by Maurelli et al. [15]. Three different PCR protocols were used for amplification of DNA samples: (i) qPCR to amplify a region of the minicircle kinetoplast DNA (kDNA) [16] was used to analyze all the DNA extracted (total = 200 DNA samples); (ii) nested PCR to amplify the small subunit ribosomal RNA (SSUrRNA) [17]; and (iii) end-point PCR to amplify the Internal Transcribed Spacer 1 (ITS-1) region [18]. These last two protocols (endpoint and nested were used to confirm positive results obtained by qPCR and to characterize the *Leishmania* species. Briefly, for qPCR, a PCR mix was prepared in a final volume of 20 μL containing 1× Bio-Rad Universal Master Mix (Bio-Rad, USA), 0.3 mM of each specific primer (LEISH-1 5′-GGCGTTCTGCGAAAACCG-3′; LEISH-2 5′-AAAATGGCATTTTCGGGCC-3′), 0.25 mM of probe (5′-FAM-TGGGTGCAGAAATCCCGTTCA-3′-BHQ1) and 2 μL of extracted DNA was prepared. Each amplification was performed in duplicate. To prepare a standard curve, a serial dilution of a positive sample, provided by the National Reference Center for Leishmaniosis (CReNaL), consisting of equivalents of DNA from 1 × 10^6^ cells to 1 cell per amplified sample, was prepared. A negative control was added for each run to verify contaminations. The thermal cycling conditions included a 10 min denaturation at 95 °C and 40 cycles of 95 °C for 15 s and 60 °C for 35 s. The reactions were performed in a CFX96 (Bio-Rad, USA). To quantify parasite burdens, cycle threshold (Ct) values obtained for each test sample were compared with those obtained for the corresponding standard curve. For SSUrRNA amplification, a first PCR mix was prepared in a final volume of 50 μL, containing 1× EmeraldAmp^®^ GT PCR mix (Takara, France), 25 pmol/ μL of each specific primer (R221 5′-GGTTCCTTTCCTGATTTACG-3′; R332 5′-GGCCGGTAAAGGCCGAATAG-3′), and 5 μL of extracted DNA. DNA samples of *Leishmania* were used as positive controls. The thermal cycling conditions included 5 min denaturation at 94 °C and 35 cycles of 94 °C for 30 s, 60 °C for 30 s, 72 °C for 30 s, and a final extension at 72 °C for 5 min. The reaction was performed in a T100 (Bio-Rad, USA). The nested PCR was prepared in a final volume of 50 μL, containing 1× EmeraldAmp^®^ GT PCR mix (Takara, France), 25 pmol/μL of each specific primer (R223 5’-TCCCATCGCAACCTCGGTT- 3′; R333 5′-AAAGCGGGCGCGGTGCTG-3′) and 5 μL of DNA amplified with the first PCR. DNA samples of *Leishmania* were used as positive controls. The thermal cycling conditions included 5 min denaturation at 94 °C and 35 cycles of 94 °C for 30 s, 65 °C for 30 s, 72 °C for 30 s, and a final extension at 72 °C for 5 min. The reaction was performed in a T100 (Bio-Rad, USA). The PCR products obtained from the nested PCR were detected on a 2% ethidium bromide-stained low melting agarose gel (Bio-Rad, USA). Bands were cut from the gel under ultraviolet (UV) exposure, and the amplified DNAs were purified by QIAquick Gel Extraction KIT (Qiagen, Germany). The purified PCR products were sequenced, and the obtained sequences, in both forward and reverse directions, were analyzed using the Chromas version 2.6.6 software and compared with sequences present in GenBank, using BLASTn system and ClustalW. For ITS-1 amplification, a PCR mix was prepared in a final volume of 50 μL, containing 1× EmeraldAmp^®^ GT PCR mix (Takara, France), 0.50 mM of each specific primer (LITSR 5′-CTGGATCATTTTCCGATG-3′; L5.8S 5′-TGATACCACTTATCGCACTT-3′) and 5 μL of extracted DNA. DNA samples of *Leishmania* were used as positive controls. The thermal cycling conditions included 4 min denaturation at 95 °C and 36 cycles of 95 °C for 40 s, 53 °C for 30 s, 72 °C for 1 min, and a final extension at 72 °C for 6 min. The reaction was performed in a T100 (Bio-Rad, USA). The PCR products were detected on a 2% ethidium bromide-stained low-melting agarose gel (Bio-Rad, USA). Bands were cut from the gel under UV exposure, and the amplified DNAs were purified by QIAquick Gel Extraction KIT (Qiagen, Germany). The purified PCR products were sequenced, and the obtained sequences, in both forward and reverse directions, were analyzed using the Chromas version 2.6.6 software and compared with sequences present in GenBank, using BLASTn system and ClustalW.

## Results

The results (Table 1) demonstrated a high percentage (21.6%) of dogs positive for at least at one or more assay and matrix, four of them (36.3%) exhibited clinical signs. The most sensitive technique was the lymph node qPCR that identified 8/11 (72.7%) positive dogs, while conjunctival swabs always resulted as negative. Clinical, serological, and molecular results are summarized in the Table 1. By qPCR, eight lymph node samples and one skin sample (correspondent to one positive lymph node sample) resulted as positive to *Leishmania* spp., with values of amastigotes/mL from 3 to 2379 for lymph nodes samples, while a value of 943,300 amastigotes/mL was obtained for skin sample. Positive samples obtained by qPCR were confirmed also by SSUrRNA and ITS-1 PCRs. By nested PCR for amplification of SSUrRNA a band of 358 bp was obtained for each positive sample. After purification of PCR products and sequencing, five lymph nodes and the positive skin sample showed an identity of 100% with *L. infantum* sequences (GenBank access number: MK495995.1), while two samples showed 99.72% of identity with *L. infantum*/*major*/*donovani* (GenBank access numbers: MK495995.1/MT560279.1/LR812647.1). By amplification of ITS-1 a band of 350 bp was obtained for each positive sample. After purification of PCR products and sequencing, five lymph nodes and the positive skin sample showed an identity of 100% with *L. infantum* sequence (GenBank access number: KM677128.1), while two samples showed an identity of 99.08% and 99.17%, respectively, with *L. major* sequences present in GenBank (access numbers: FJ753395.1/MN604136.1). The new two sequences have been registered in GenBank with Access numbers: PP534960 and PP536550. Serology conventionally used to detect antibodies against *L. infantum* showed positive results in 6/11 dogs, while negative results were observed in the two dogs infected by *L. major*.

**Table 1:** Clinical, serological, and molecular results of *Leishmania* spp. infected dogs, in Echrarda area, Tunisia

Dog code	Age (Years)	Sex	Clinical signs	Quantitative serology	BC PCR	L PCR	C PCR	S PCR	4Dx	*Leishmania* species
K6	1.5	M	Le	−	−	+	−	−	AE	*L. major*
K23	11	F	−	−	−	+	−	−	A	*L. infantum*
K28	5.5	F	Le	−	−	+	−	−	A	*L. major*
K32	1.5	F	−	−	−	+	−	−	A	*L. infantum*
K37	4.5	M	−	−	−	+	−	−	ADE	*L. infantum*
K38	3.5	F	WL	+	+	−	−	−	−	*Leishmania spp*.
K40	3.5	M	Le	+	+	−	−	−	AE	*Leishmania* spp.
K47	3	F	−	+	+	+	−	−	D	*L. infantum*
K49	1.5	F	−	+	−	−	−	−	A	*Leishmania spp.*
K50	3	F	−	+	−	+	−	+	−	*L. infantum*
K51	1	M	−	+	+	+	−	−	−	*L. infantum*

*BC* buffy coat, *L* lymph node aspirate, *C* conjunctival swab, *S* skin punch biopsy, *Le* lymph node enlargement, *WL* weigh loss, *A* positive for *Anaplasma* spp., *E* positive for *Ehrlichia canis*, *D* positive for *Dirofilaria immitis*

IDEXX 4× rapid test identified eight dogs co-infected with other CVBDs, the most frequent being *Anaplasma* spp. that was present in both *L. major*-positive dogs, followed by *Ehrlichia canis* and *Dirofilaria immitis.*. One dog was infected by four different pathogens. Interestingly, one dog infected by *L. major* was living in the same house where a 6-year-old child showed cutaneous lesions referred to as ZCL.

## Discussion

Among neglected tropical diseases (NTDs), cutaneous leishmaniasis (CL) represent one of the most important sanitary problems in many countries of the world, affecting millions of people each year, in which cutaneous disfiguring effects may result after the infection occur. ZCL causes thousands of new clinical cases in Tunisia, with the population of the governorates of Kairouan, Sidi Bouzid, and Gafsa, representing the 87% of the total population at risk [19]. Wild animal reservoirs are considered as a major source of parasite’s transmission to maintain the zoonotic cycle of *L. major.*. Sand fly and rodent reservoirs control programs have not reached the desirable result due to the geographical context, semi-arid large environment that are very difficult to cover, and the animal behavior characterized by the digging of burrows with many entrances. Additionally, many factors may amplify the presence of the transmitting insects and rodents, the main being the inadequate household garbage disposal around the rural houses of this area. In this social context with low economical resources, the dogs are mostly bread as guard dogs living outdoors, exposed to the same sand fly-biting risk as humans. Canine reservoirs are well accepted as a main source of zoonotic transmission to maintain peri-urban and rural *L. infantum* infection [20]. Domestic dogs were also found infected by *L. infantum* in a neighboring ZCL endemic area, as assessed by a previous study that evidenced a lower prevalence of infection when compared with endemic well-established foci of canine and human infections [6]. Our results confirm that also in endemic ZCL areas, the presence of *L. infantum* parasite can result in high prevalence of infection in dogs, indicating the dog as the most sensible host to this *Leishmania* species. Many factors can contribute to the establishment and progression of *L. infantum* infection; the severity of late-stage disease is correlated with the decrease of the cellular immunity, high antibody levels, and increasing parasite load. Several studies have demonstrated that dogs exposed to tick-borne co-infections have a higher relative risk of progression to clinical leishmaniosis. Dogs with canine leishmaniosis (CanL) and co-infections with either *Ehrlichia canis, Babesia canis,*, and *Rickettsia conorii* had a shorter survival time [21]. Additionally, Toepp et al [22]. It was found that dogs with multiple tick-borne co-infections had a statistically significant increased risk for progression of CanL and increased risk for mortality. The high prevalence of single or multiple tick-transmitted infections together with the low sanitary management of the *Leishmania* spp. infected dogs found in the present study, confirms the establishment of *Leishmania* infection as a contribution of many immune unbalancing factors. The detection of mild clinical picture of the infected dogs is probably due to the young average age of the enrolled dogs; however, it was not possible to assess the definitive severity of the disease with additional hematological and biochemical parameters. The two dogs that were found to be infected with *L. major* exhibited mild clinical signs and both showed antibodies against tick-transmitted infection, *Anaplasma* and *Ehrlichia* spp. The contribution of these arthropod-borne bacterial infections for the establishment of *L. major* infection has never been studied, but their role could be very similar to what happens in dogs infected with *L. infantum*. No cutaneous lesions were detected in these dogs, where negative skin PCR in both animals may suggest how the skin was not the most relevant infected tissue. Interestingly the parasite’s DNA was found in the popliteal lymph node of both dogs. The presence of *L. major* in the lymph node has been demonstrated [8] in a 6-month young dog with cutaneous manifestation on the muzzle. These findings can suggest a possible visceral spread of a cutaneous *Leishmania* species in dogs. Interestingly, one of the two positive dogs of the present study was young, with an estimated age of 1.5 year, similar to another young puppy that was found clinically sick in Israel [9]. Due to the limited number of cases, it is not possible to have a clear correlation between age and infection, in addition to the limited life expectation of dogs living in this difficult socio-economical context. Diagnosis of *L. major* infection in dogs is not easy due to the limited availability of serological tests. ELISA and IFAT serology, which use the whole *Leishmania* promastigote antigen, do not allow to distinguish among different *Leishmania* species. In addition, dogs infected with *L. major* are negative to rK39 antigen kit, while dogs infected with *L. infantum* and *L. tropica* tested positive [9]. The definitive diagnosis of *L. major* infection in dogs is performed by PCR with DNA sequencing; this finding complicates studies on large number of dogs. In the present study the detection of *Leishmania* spp. has been performed on different sampled tissues. It is known that many variables can affect the PCR sensitivity as the examined tissues and the amplified DNA target number of copies. Bone marrow, lymph node, spleen, skin, and conjunctival swabs are the best materials used for detection of *Leishmania* DNA, with PCR targeting kinetoplast DNA (kDNA) being the most used assay [23]. Moreover, high sensitivity of real-time PCR targeting kinetoplastic DNA in detecting *L. major* during zoonotic CL lesions has been reported [24].

## Conclusions

The epidemiological role of dog for this *L. major* transmission remains under investigation, due to the limited number of diagnoses, and the absence of knowledge on the infectiousness of the healthy infected dogs, the epidemiological role of which is well assessed for *L. infantum*. Interestingly, one dog that tested positive for *L. major* was living in the same house where a 6-year-old child showed cutaneous lesions referred to ZCL; however, we had not the possibility to perform adjunctive epidemiological investigations The recent demonstration of eight *L. major* canine cases in a survey performed in Burkina Faso, where *L. major* is considered endemic for humans, amplifies the need of knowledge on the role of dog as reservoir [13]. Undoubtably, the development of urbanized areas in endemic ZCL rural contexts may contribute to the change of rodents’ natural habitats, with an increased possibility to have the dog as a major blood source for transmitting sand flies and to consider it a potential reservoir of the *L. major* parasite.

## Data Availability

The data supporting the findings of the study are available within the article.
